# HPTLC Analysis of *Solanum xanthocarpum* Schrad. and Wendl., a *Siddha* Medicinal Herb

**DOI:** 10.1155/2018/8546306

**Published:** 2018-11-08

**Authors:** Raman Preet, Raghbir Chand Gupta

**Affiliations:** Department of Botany, Punjabi University, Patiala, Punjab, India

## Abstract

In the present study, HPTLC is used to detect the presence and amount of triterpenoids and phytosterols in different plant parts (fruit, stem, leaf, and root) of *Solanum xanthocarpum* Schrad. and Wendl.; such analysis is done for the first time. Each plant part has its own medicinal value and is used as *Siddha* medicinal herb. The employed statistical analysis ensures that the developed method is reproducible and selective. The results show that the fruit samples contain highest amount of tested phytochemicals. This method can be used as an important tool to ensure the therapeutic dose in herbal formulations, standardization, and quality control of bulk drugs.

## 1. Introduction

*Solanum xanthocarpum* Schrad. and Wendl., commonly known as *Kantkari*, belongs to family Solanaceae. It comprises 90 genera and 2000–3000 species. It is distributed to plains and lower hills of India. It is an herbaceous spiny perennial herb with prominent nodes and internodes. Roots are almost cylindrical and tapering. Flowers are purple colored and few flower axillary cymes with glabrous, globular berry are green when young and turn yellow at maturity. The seeds are smooth, compressed, and reniform with bitter taste. It is known for its traditional medical value, and recent scientific studies have emphasized the possible use of this plant in the modern medicine system. India is rich with its biodiversity and knowledge of rich ancient traditional systems of medicine like Ayurveda, Siddha, Unani, Amchi, and local health traditions [1]. Wild plants serve as an indispensable constituent of the human diet. They supply with minerals, vitamins, protein, and certain hormone precursors [2–4]. Roots, stem, leaves, flowers, and fruits are useful parts of this herb as *siddha* medicinal herb [5]. However, there is need to study the inexpensive nutritive value of these wild plants so that these can be exploited for their pharmaceutical preparations.

## 2. Experimental

At present triterpenoids and phytosterols are separated from methanolic extracts in different plant parts (fruit, stem, leaf, and root) in *S. xanthocarpum* by using high-performance thin layer chromatography (HPTLC). Details of qualification and quantification of different mobile phases used are mentioned in Table 1. Part-based separation of bioactive compounds from the wild samples is done first.

### 2.1. Plant Material

Plant samples were collected from different localities of Indian Thar desert, Rajasthan. The specimens were collected and deposited in the herbarium of Punjabi University, Patiala, with accession numbers 59194 and 59931. The plants parts, i.e., fruit, leaf, stem, and root samples, were separated washed and dried at room temperature (25°C–30°C).

### 2.2. Stock Solution

All the organic solvents as well standards of analytical grade used in the present study were purchased from Merck (Darmstadt, Germany). Solutions of standards were prepared by using 1 : 1 methanol.

### 2.3. Apparatus

For the acquisition, a Camag HPTLC system comprising a Linomat-V automatic sample applicator and Camag TLC scanner III with win CATS 4 software for interpretation of data was used. A Camag 100 *µ*L precision syringe from Hamilton, Bonaduz, Switzerland, was used for sample application under gentle stream of nitrogen. Camag aluminum precoated silica gel 60-_F254_ plates with 200 *µ*m thickness × 5 *µ*m particle size from Merck (Darmstadt, Germany) was used. For the plate development, a Camag twin-trough chamber 20 cm *W* × 10 cm *H* was used.

### 2.4. Chromatography

Chromatographic studies were performed using the following conditions: HPTLC was carried out using aluminum plates precoated with silica gel 60F_254_. Combinations of different mobile phases were used to quantify different standards (Table 1); volume of mobile phase was kept up to 20 mL; chamber saturation time: 30 min; temperature: 25 + 18°C; relative humidity: 35%–40%; migration distance: 80 mm; migration time: 30 min; wavelength of detection (Table 1); scanning speed: 20 mm/s; data resolution: 100 mm/step; and band width: 4mm. A Camag video documentation system was used for imaging and archiving the thin layer chromatograms. The object was captured by means of a highly sensitive digital camera. Image acquisition processing and archiving were controlled via Win CATS software.

### 2.5. Preparation of Derivative Reagent

Anisaldehyde sulphuric acid was prepared by dissolving 5 mL of *p*-anisaldehyde solution in 1 ml of 98% sulphuric acid and 50 ml of acetic acid. After development and derivatization of the plate, measurements were made by winCATS software. Concentration of the target analytes in the separated bands was determined from the intensity of the reflected light indicated and the peak areas produced were correlated to the analyte concentrations using six-level linear calibration curves.

### 2.6. Chromatographic Separation

**Figure 1:**
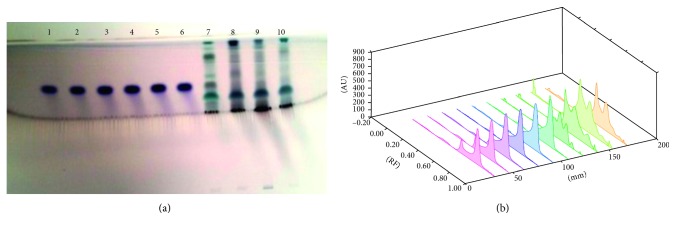
(a) HPTLC fingerprint profile of lupeol (tracks 1–6) in fruit (track 7); leaf (track 8); stem (track 9); and root (track 10) of *S. xanthocarpum*; (b) 3D view of densitogram at 530 nm.

**Figure 2 fig2:**
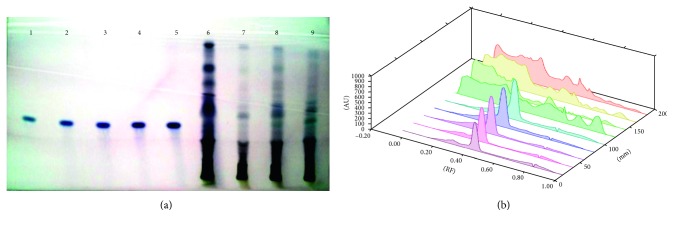
(a) HPTLC fingerprint profile of oleanolic acid (tracks 1–5) in fruit (track 6); leaf (track 7); stem (track 8); and root (track 9) of *S. xanthocarpum*; (b) 3D view of densitogram at 530 nm.

**Figure 3 fig3:**
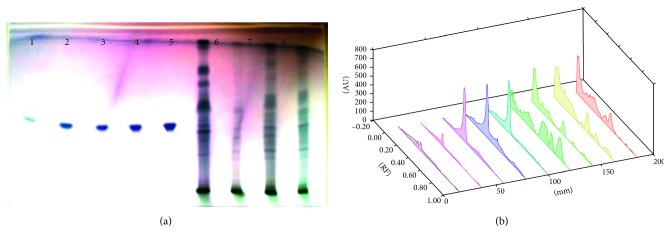
(a) HPTLC fingerprint profile of ursolic acid (tracks 1–5) in fruit (track 6); leaf (track 7); stem (track 8); and root (track 9) of *S. xanthocarpum*; (b) 3D view of densitogram at 510 nm.

**Figure 4 fig4:**
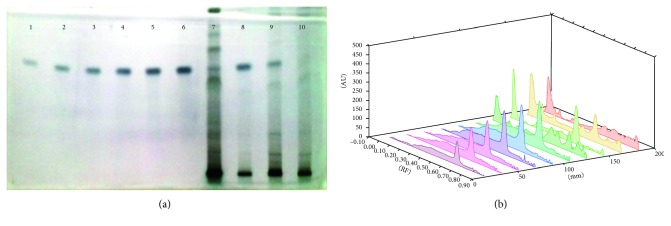
(a) HPTLC fingerprint profile of *β*-sitosterol (tracks 1–6) in fruit (track 7); leaf (track 8); stem (track 9); and root (track 10) of *S. xanthocarpum*; (b) 3D view of densitogram at 530 nm.

**Figure 5 fig5:**
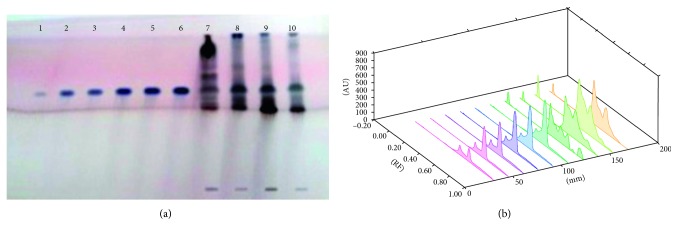
(a) HPTLC fingerprint profile of campesterol (tracks 1–6) in fruit (track 7); leaf (track 8); stem (track 9); and root (track 10) of *S. xanthocarpum*; (b) 3D view of densitogram at 530 nm.

**Figure 6 fig6:**
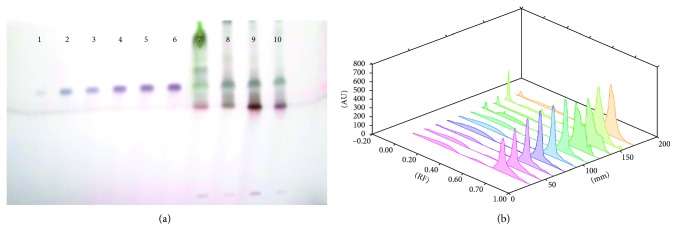
(a) HPTLC fingerprint profile of ergosterol (tracks 1–6) in fruit (track 7); leaf (track 8); stem (track 9); and root (track 10) of *S. xanthocarpum*; (b) 3D view of densitogram at 530 nm.

**Figure 7:**
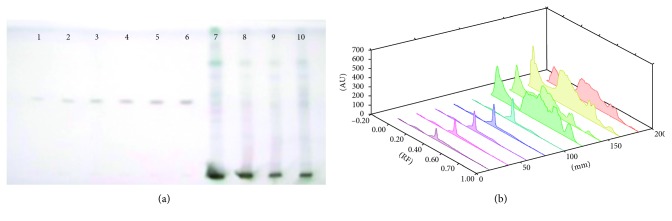
(a) HPTLC fingerprint profile of withanolide B (tracks 1–6) in fruit (track 7); leaf (track 8); stem (track 9); and root (track 10) of *S. xanthocarpum*; (b) 3D view of densitogram at 530 nm.

Each extract of 5 *µ*L *S. xanthocarpum* solution was spotted on the HPTLC silica gel plate, 4 mm band length, using a Camag ATS4 automatic TLC sampler spotting device. The TLC plate was developed in the ascending mode in a twin-trough chamber presaturated for 30 mins with particular mobile phase. Linear ascending plate development was performed until a migration of distance 8 cm from the origin was reached. The plate was removed from the chamber, air dried, derivation with *p*-ansaldehyde sulphuric acid, heated, and scanned in the absorbance/reflectance mode of a Camag TLC scanner 3 (Figures 1 [Fig fig2] [Fig fig3] [Fig fig4] [Fig fig5] [Fig fig6]–7). Peak area data were recorded using Camag Win CATS software.

### 2.7. Calibration Curve

A standard solution volume of 2–10 *µ*L of all the analyzed sugars was used. Concentration of the target analysts in the separated bands were determined from the intensity of the reflected light indicated, and the produced peak areas were correlated to the analyst concentrations using six-level linear calibration curves. The employed statistical analysis ensures that the developed method is reproducible and selective. This method can be used as an important tool to ensure the therapeutic dose in herbal formulations, standardization, and quality control of bulk drugs.

## 3. Validation of HPTLC Densitometry Method Specificity

### 3.1. Specificity

The specificity of the method was ascertained by analyzing standard compounds and samples. The spots for standards in samples were confirmed by comparing the R*f* and spectra of the spots with that of the standards. The peak purity of all standards were assessed by comparing the spectra at three different levels, i.e., peak start, peak apex, and peak end positions of the spot.

### 3.2. Precision

To define deviations due to the instrument, six different samples of the same were spotted on HPTLC silica gel plates and analyzed to determine variations arising due to method itself (Table 1).

### 3.3. Recovery

The recovery of the method was determined at two levels, i.e., 50% and 100%, by adding a known amount of particular standard to the extracts of plant part, and the mixtures were analyzed by the proposed method.

### 3.4. Ruggedness

The ruggedness of the proposed method was studied using reagents from different lots and different manufacturers.

### 3.5. Limit of Detection and Limit of Quantitation

The limit of detection (LOD) and limit of quantitation (LOQ) were determined, and data pertaining to LOD, LOQ, interday, and intraday precision are given in Table 1. The significant difference between the amounts of particular compound in each plant is also mentioned in Table 3.

### 3.6. Sample Preparation

Plant parts like fruit, leaf, stem, and root of the plants were extracted with methanol by using the Soxhlet apparatus. The plant material was shade-dried and coarsely powdered before Soxhlet apparatus application. 10 g of each dried and powdered aerial plant parts was applied to the methanolic extraction independently in the Soxhlet apparatus. The extracts were concentrated using a rota-evaporator and then lyophilized. Powdered extracts was weighed, and 5 mg of each was dissolved in 5 mL of methanol to obtain 1 mg/1 mL concentration.

## 4. Results

Plant samples were collected from different localities of Indian Thar desert, Rajasthan. Present studies reveal unequal concentration of bioactive compounds in different plant parts. This phenomenon is very common in many secondary metabolities [6, 7].

Different solvents are examined for the separation of these bioactive compounds, and the best combinations for separation are listed in Tables 1 and 2. Triterpenoids detected are lupeol, oleanolic acid, and ursolic acid. The fruit (6.81 ± 0.23 *µ*g/mg DWE) and stem (6.179 ± 0.61 *µ*g/mg DWE) samples of the plant are rich in lupeol content. It is completely absent in leaf samples and present in very less amount in root samples (Figure 1; Table 3). Roots are found to be quite rich in oleanolic acid (24.67 ± 0.582 *µ*g/mg DWE) and ursolic acid (8.48 ± 0.31 *µ*g/mg DWE) followed by stem samples (6.39 ± 0.97 *µ*g/mg DWE; 1.07 ± 0.19 *µ*g/mg DWE), (Figures 2 and 3; Table 3). Among the triterpenoids, earlier oleanolic acid was isolated using paper, thin layer, and column chromatography [8]. There is no earlier report of part-based isolation or separation of these triterpenoids by using HPTLC in wild samples of the plant.

Amount of *β*-sitosterol is reported to be high in stem samples of the plant (20.85 ± 0.96 *µ*g/mg DWE) followed by the leaf samples (19.89 ± 1.53 *µ*g/mg DWE) and root samples (8.04 ± 0.055 *µ*g/mg DWE). The least amount of *β*-sitosterol was reported in fruits (6.42 ± 0.91 *µ*g/mg DWE) of the plant (Figure 4). The root samples are quite rich in phytosterodial composition (28.19 ± 0.01 *µ*g/mg DW) of campesterol and ergosterol (24.27 ± 0.28 *µ*g/mg DW; Figures 5 and 6). Withaferin A and withanolide A and B were also tested for their presence. But only withanolide B was present. Withanolide B is also separated along with other bioactive compounds (Tables 1 and 2). It is present only in the fruit (17.59 ± 0.12 *µ*g/mg DWE) and the root samples (10.09 ± 0.14 *µ*g/mg DWE) of the plant (Figure 7). Phytosterols are the most important constituents which increases the medicinal value of the plant. Earlier, there was no report of determination of different bioactive compounds from the plant by using HPTLC. At present, the methanolic extract of the plant are used to separate different bioactive compounds (Tables 1 and 2). The plant is reported to be very rich in phytosteroidal content (Table 3).

Among triterpinoids, earliar oleanolic acid was isolated using paper, thin layer, and column chromatography [9], and lupeol was reported in fruits in tissue cultures of *solanum xanthocarpum* [10]. Phytosterols are the most important constituents which increases its medicinal value of the plant. Heble et al. [11] reported the presence of *β*-sitosterol through tissue culture techniques. The plant was estimated for its fatty acids content in [12].

## 5. Discussion

A simple, rapid, reliable method is developed and validated for the qualitative and quantitative determination of different phytochemicals in plant matrices. A significant difference was obtained among the different plant parts. The highest amount of most of the compounds was noted in fruit samples of the plant. The results clearly show that the fruits are the very supplier of different phytochemicals mainly phytosterols and should be explored more in the production of medicinal drugs in pharmaceutical companies.

## Figures and Tables

**Table 1:** Data showing bioactive markers, composition of solvent system, derivatizing reagent used and wavelength of all the marker compounds used in the present study for HPTLC analysis.

S. no.	Bioactive compound	Solvent system	Composition (v/v/v/v)	Derivatizing reagent	Wavelength (nm)
*Triterpenoids*					
1.	Lupeol	Toluene:methanol:formic acid	9 : 4: 0.2	*p*-Anisaldehyde sulphuric acid	530
2.	Oleanolic acid	Toluene: ethylacetate:formic acid	7 : 3 : 0.3	*p*-Anisaldehyde sulphuric acid	540
3.	Ursolic acid	Toluene: ethylacetate:formic acid	8 : 2 : 0.1	*p*-Anisaldehyde sulphuric acid	510
*Phytosterols*					
4.	*β*-Sitosterol	Toluene: ethylacetate	9 : 4	*p*-Anisaldehyde sulphuric acid	530
5.	Campesterol	Toluene:methanol:formic acid	9 : 4 : 0.2	*p*-Anisaldehyde sulphuric acid	530
6.	Ergosterol	Toluene:methanol:formic acid	9 : 4 : 0.2	*p*-Anisaldehyde sulphuric acid	530
7.	Withanolide B	Toluene:methanol:formic acid	9 : 4 : 0.2	*p*-Anisaldehyde sulphuric acid	530

**Table 2:** Data showing different parameters analyzed for the reference compounds during the present study by using HPTLC.

Reference compound	Lupeol	Oleanolic acid	Ursolic acid	*β*-Sitosterol	Campesterol	Ergosterol	Withanolide B
Working concentration (*µ*g/band)	2–10	2–10	2–10	2–10	2–10	2–10	2–10
R*f* value	0.84	0.47	0.36	0.64	0.74	0.90	0.64
Regression equation	*Y* = 2653 ∗ *X* + 1548	*Y* = 3621 ∗ X − 969.3	*Y* = 924.3 ∗ X − 155.1	*Y* = 1308 ∗ *X* + 609.7	*Y* = 2554 ∗ *X* + 214.4	*Y* = 1652 ∗ *X* + 348.7	*Y* = 470 ∗ *X* + 319.4
Correlation coefficient (*r* ^*2*^)	0.998	0.997	0.997	0.995	0.993	0.995	0.995
LOD (ng)	407	304	578	419	928	527	488
LOQ (ng)	1234	921	987	1272	2813	1598	1479

**Table 3:** Phytochemical studies in different plant parts of selected marker compounds in *S. xanthocarpum* by using HPTLC.

Bioactive compound	Fruit (*µ*g/mg)	Stem (*µ*g/mg)	Leaf (*µ*g/mg)	Root (*µ*g/mg)
Chlorogenic acid	20.86 ± 0.25	7.75 ± 0.47	0.37 ± 0.07	0.90 ± 0.06
Apigenin	2.95 ± 0.36	6.57 ± 0.32	6.61 ± 0.76	10.12 ± 0.65
Lupeol	6.81 ± 0.23	0.17 ± 0.61	Nd	1.73 ± 0.14
Oleanolic acid	16.43 ± 0.66	6.39 ± 0.97	17.98 ± 0.67	24.67 ± 0.58
Ursolic acid	5.45 ± 0.24	11.07 ± 0.19	8.64 ± 0.16	8.48 ± 0.31
*β*-Sitosterol	6.42 ± 0.91	20.85 ± 0.96	19.89 ± 1.53	8.049 ± 1.05
Campesterol	26.73 ± 0.004	14.34 ± 0.95	13.86 ± 0.43	28.19 ± 0.018
Ergosterol	12.09 ± 0.40	9.35 ± 0.32	12.09 ± 0.48	24.27 ± 0.28
Withanolide B	1.95 ± 0.068	8.29 ± 0.37	3.43 ± 0.072	34.09 ± 0.53
Emodin	Nd	0.82 ± 0.40	1.01 ± 0.74	0.61 ± 0.04

## Data Availability

The data used to support the findings of this study are available from the corresponding author upon request.
